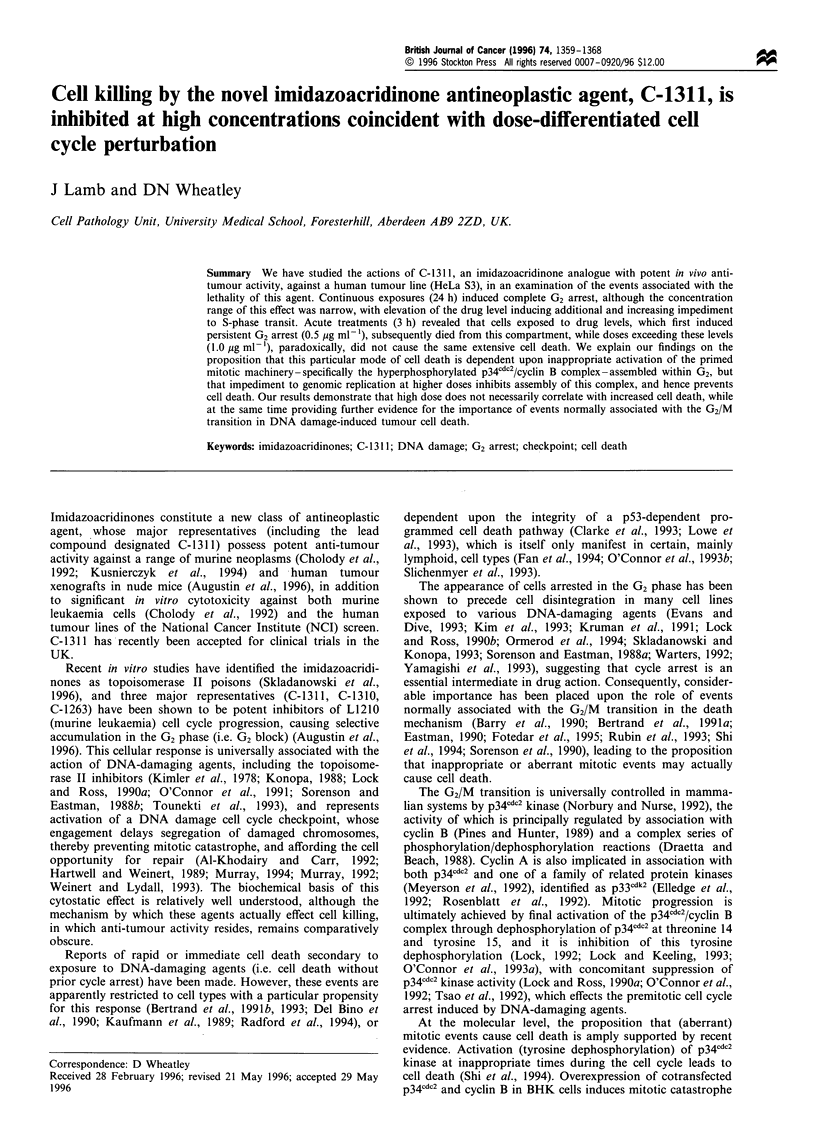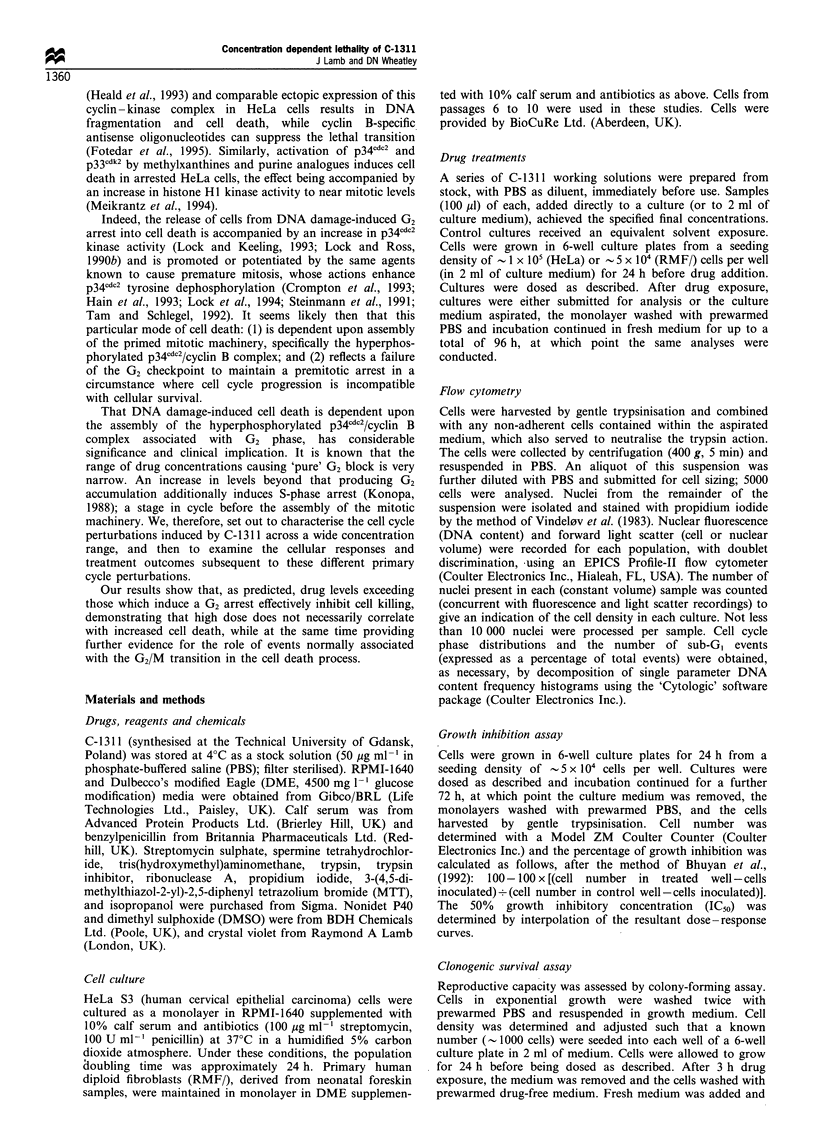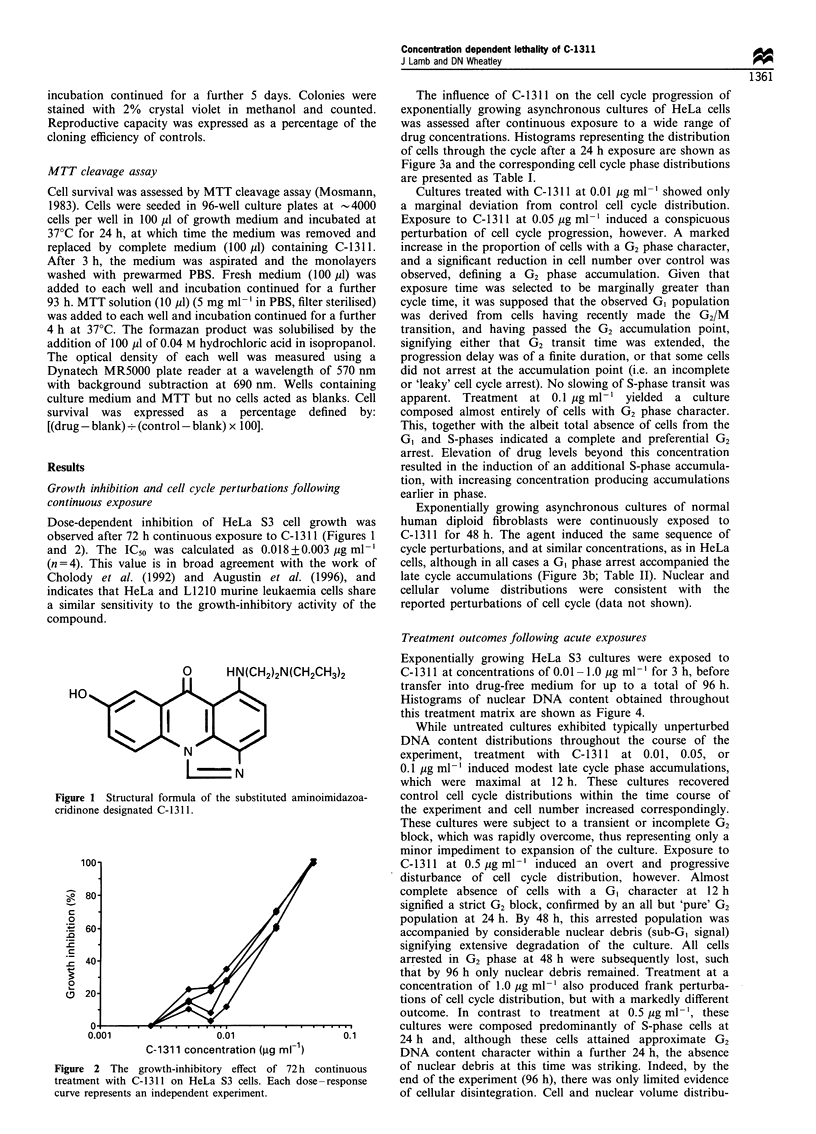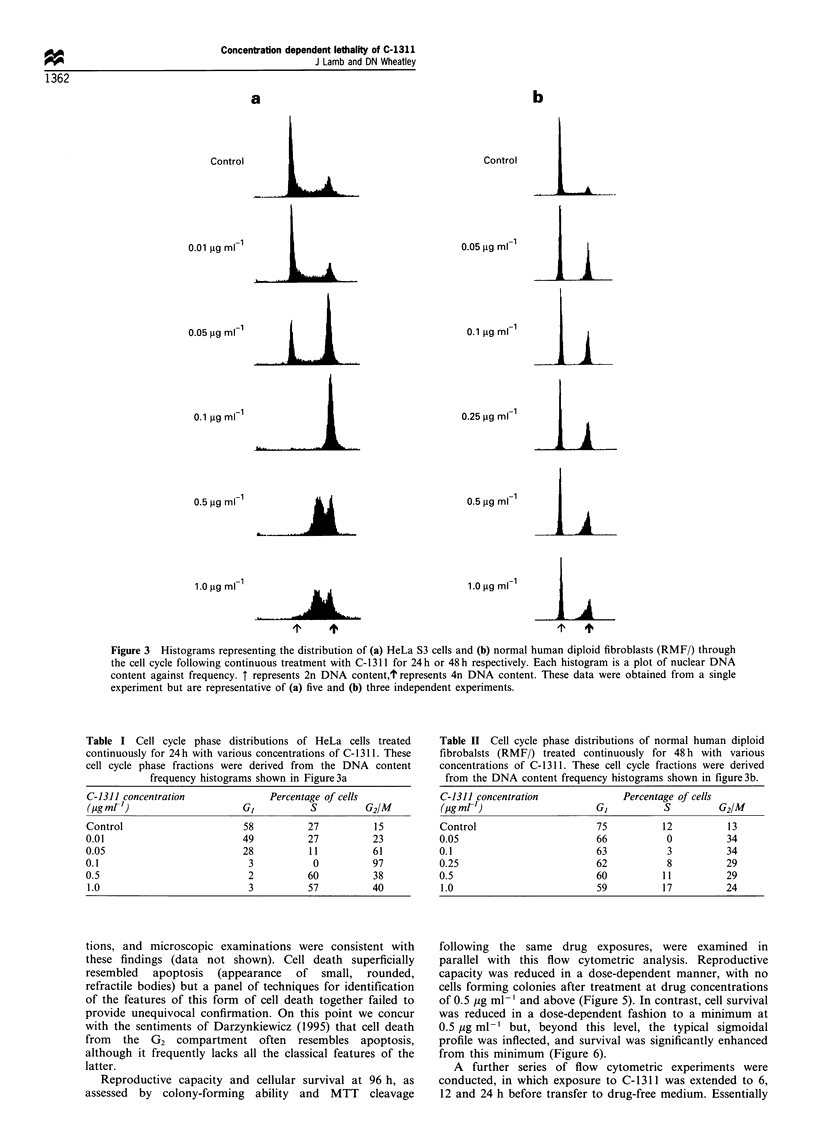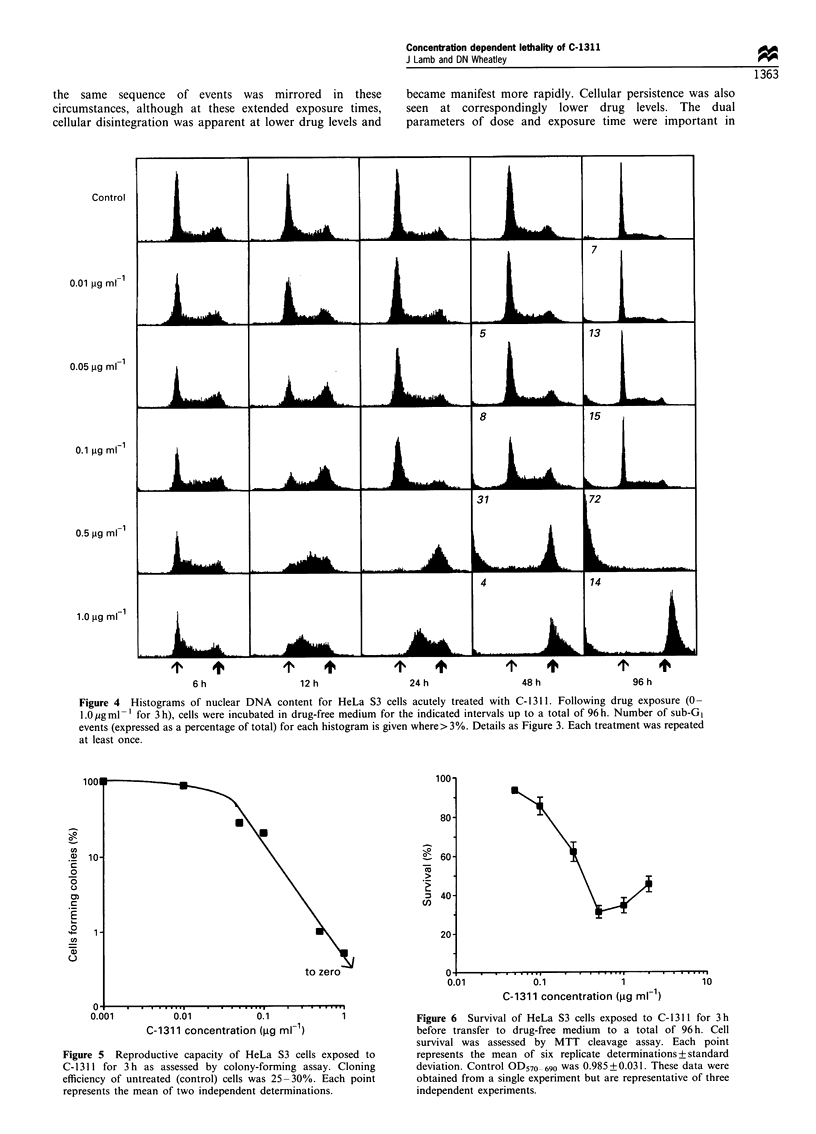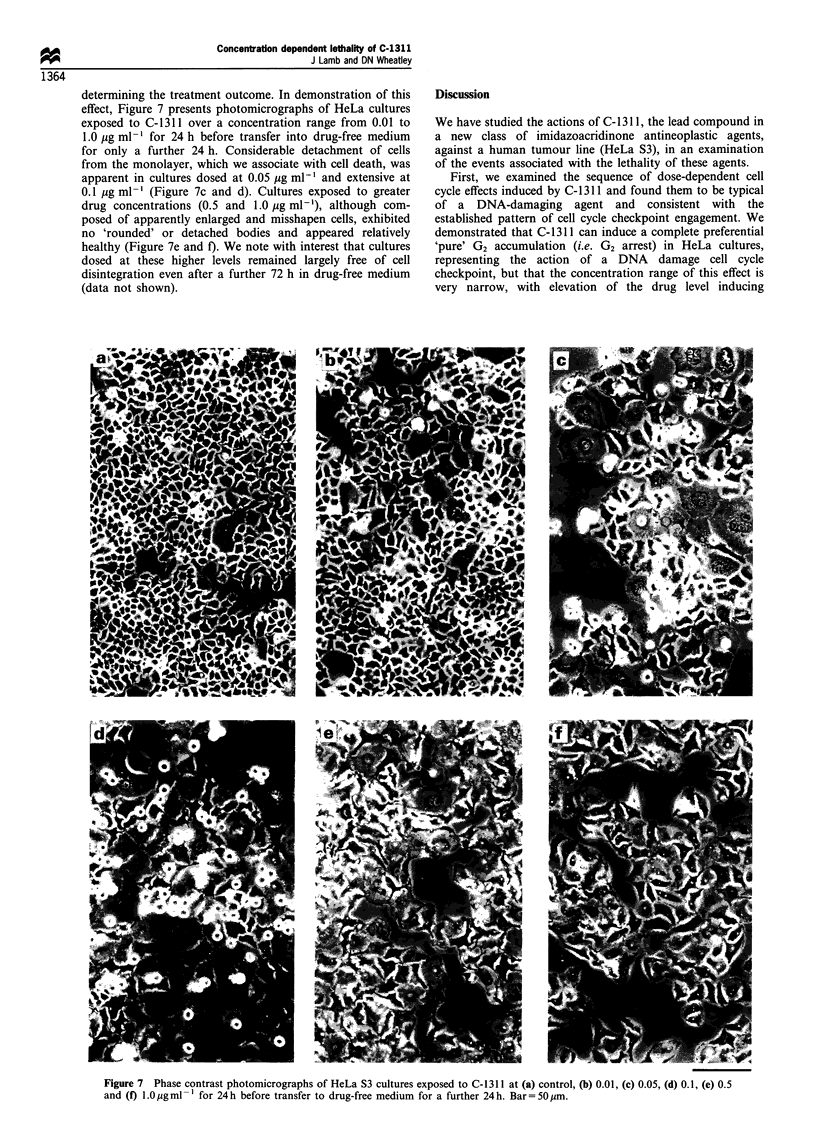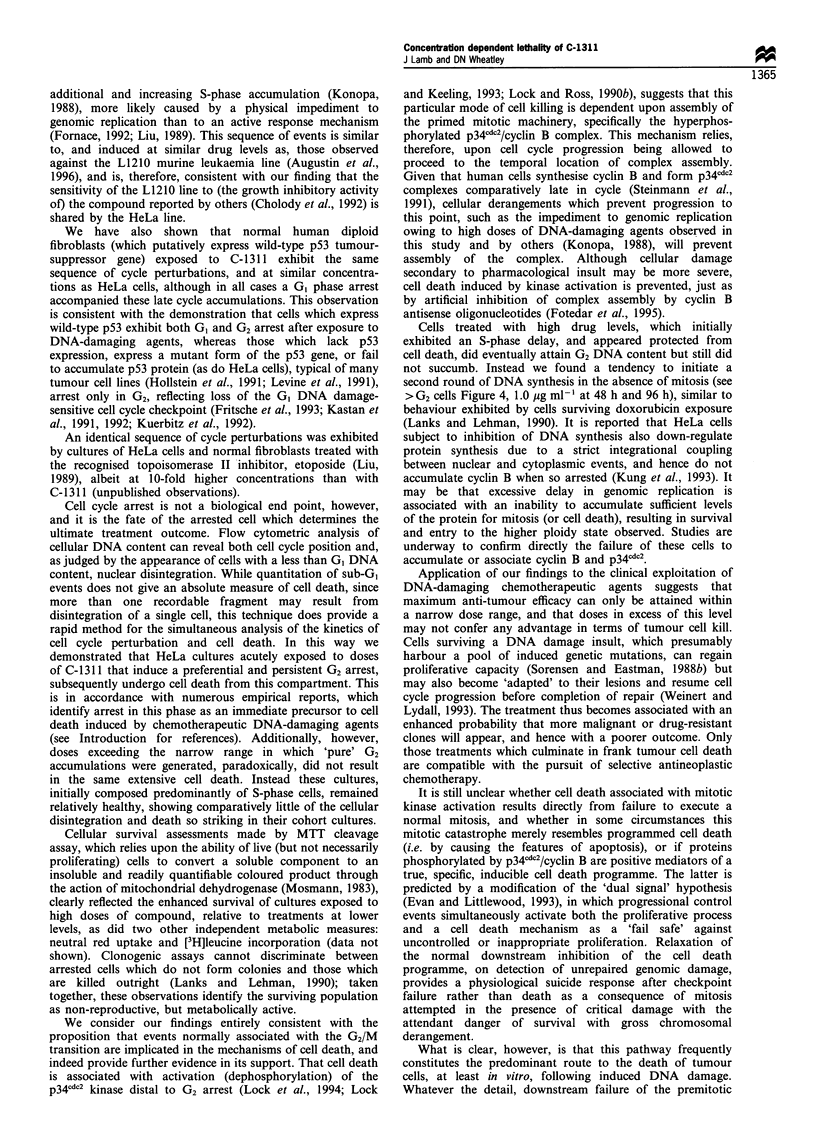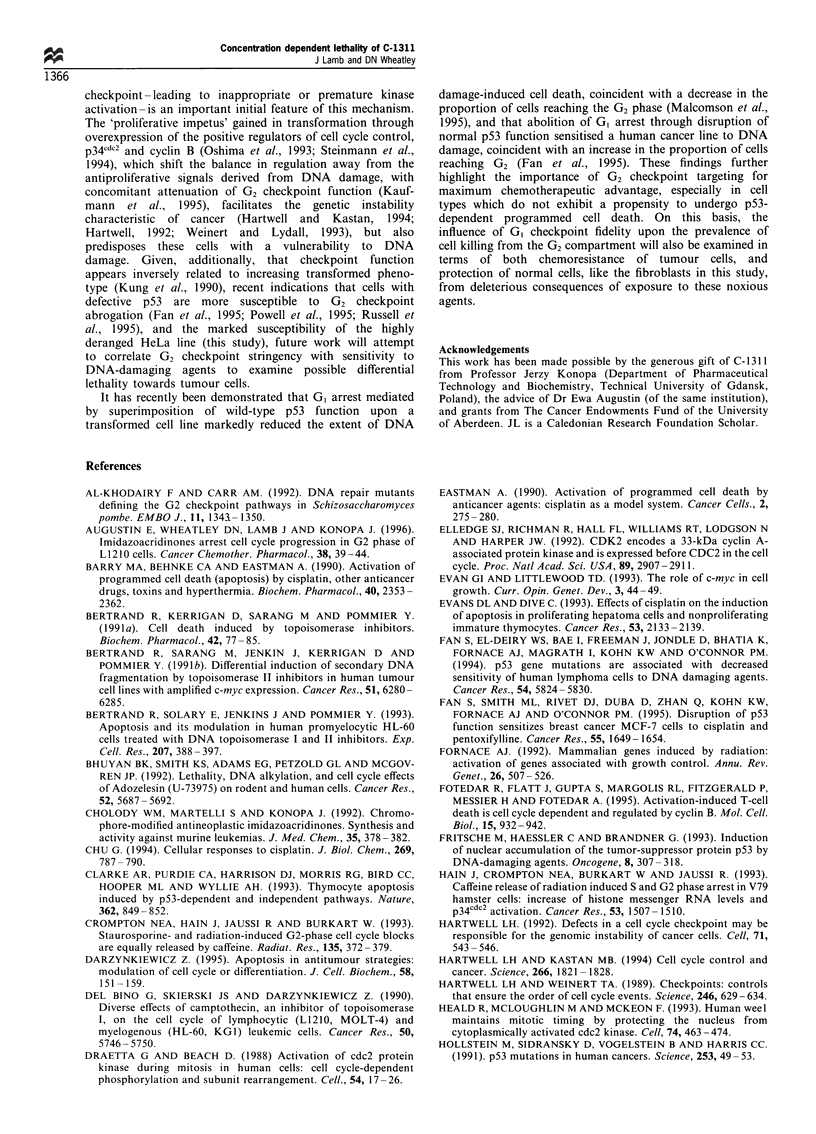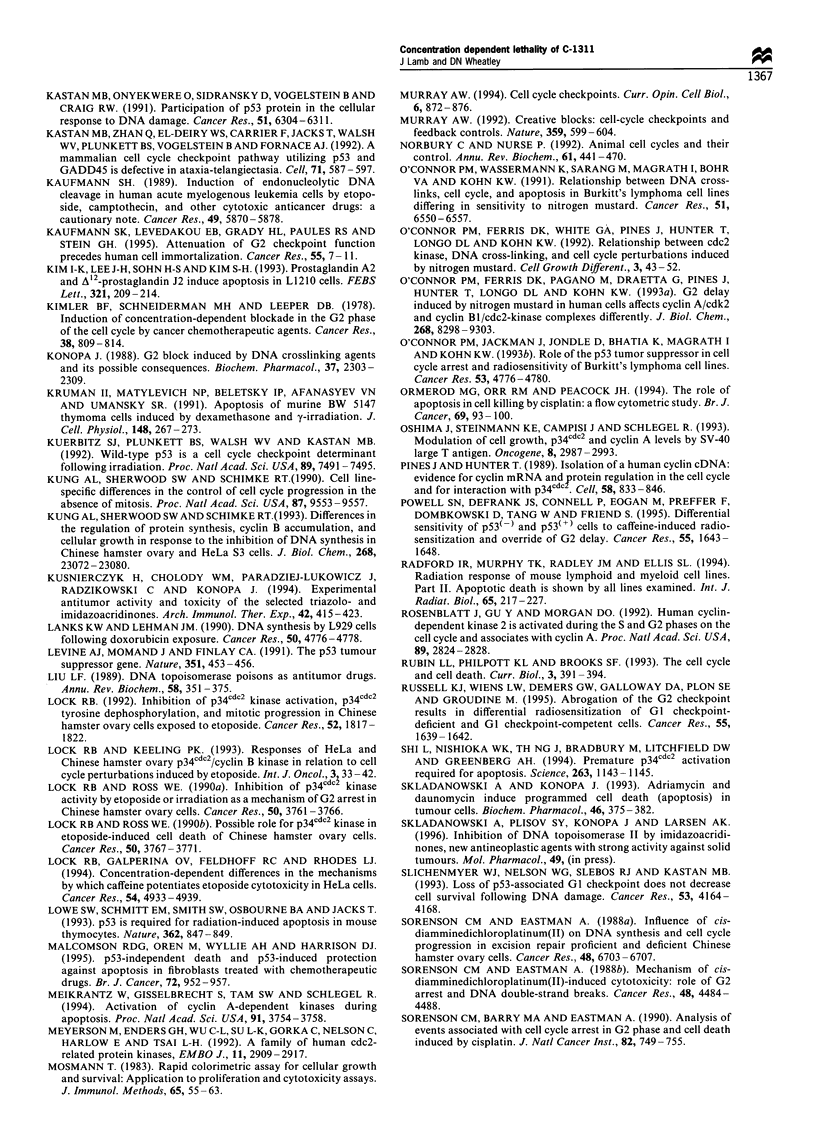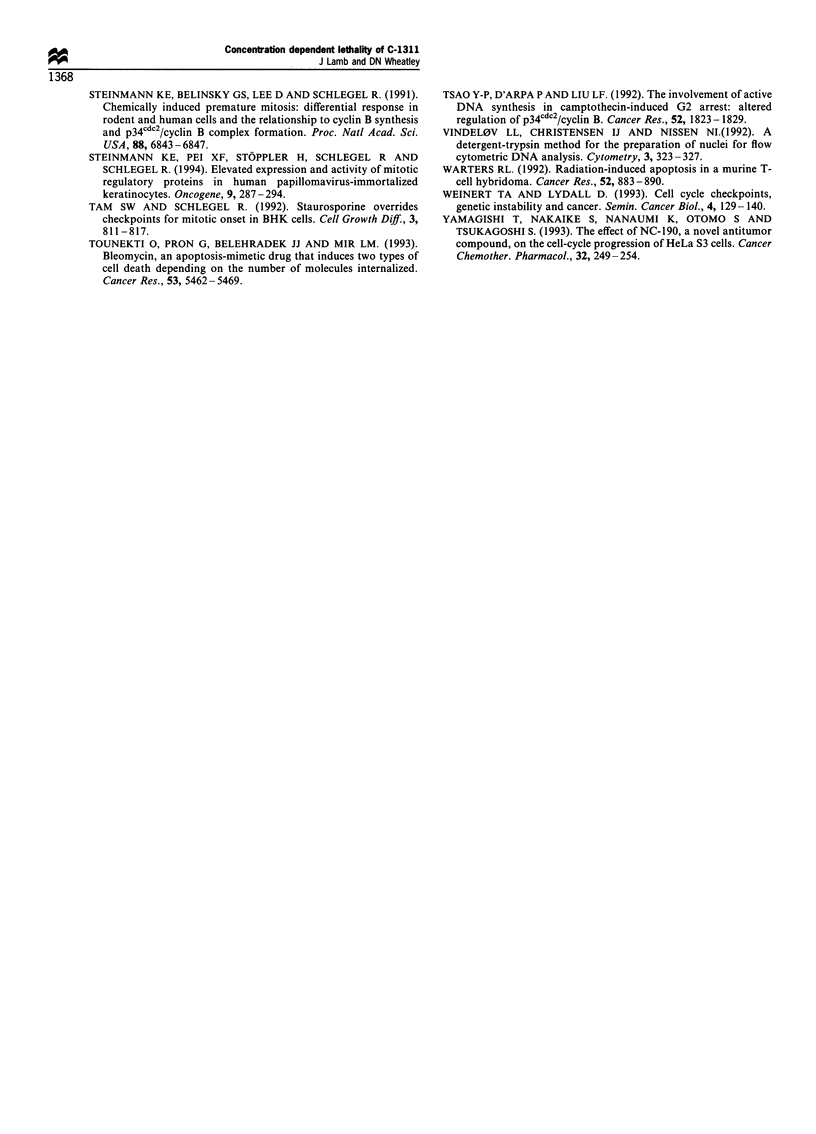# Cell killing by the novel imidazoacridinone antineoplastic agent, C-1311, is inhibited at high concentrations coincident with dose-differentiated cell cycle perturbation.

**DOI:** 10.1038/bjc.1996.550

**Published:** 1996-11

**Authors:** J. Lamb, D. N. Wheatley

**Affiliations:** Cell Pathology Unit, University Medical School, Foresterhill, Aberdeen, UK.

## Abstract

**Images:**


British Journal of Cancer (1996) 74, 1359-1368
© 1996 Stockton Press All rights reserved 0007-0920/96 $12.00

### Cell killing by the novel imidazoacridinone antineoplastic agent, C-1311, is inhibited at high concentrations coincident with dose-differentiated cell cycle perturbation

J Lamb and DN Wheatley

*Cell Pathology Unit, University Medical School, Foresterhill, Aberdeen AB9 2ZD, UK.*

**Summary** We have studied the actions of C-1311, an imidazoacridinone analogue with potent *in vivo* anti-tumour activity, against a human tumour line (HeLa S3), in an examination of the events associated with the lethality of this agent. Continuous exposures (24 h) induced complete $G_2$ arrest, although the concentration range of this effect was narrow, with elevation of the drug level inducing additional and increasing impediment to S-phase transit. Acute treatments (3 h) revealed that cells exposed to drug levels, which first induced persistent $G_2$ arrest (0.5 $\mu$g ml$^{-1}$), subsequently died from this compartment, while doses exceeding these levels (1.0 $\mu$g ml$^{-1}$), paradoxically, did not cause the same extensive cell death. We explain our findings on the proposition that this particular mode of cell death is dependent upon inappropriate activation of the primed mitotic machinery–specifically the hyperphosphorylated $p34^{cdc2}$/cyclin B complex–assembled within $G_2$, but that impediment to genomic replication at higher doses inhibits assembly of this complex, and hence prevents cell death. Our results demonstrate that high dose does not necessarily correlate with increased cell death, while at the same time providing further evidence for the importance of events normally associated with the $G_2$/M transition in DNA damage-induced tumour cell death.

**Keywords:** imidazoacridinones; C-1311; DNA damage; $G_2$ arrest; checkpoint; cell death

Imidazoacridinones constitute a new class of antineoplastic agent, whose major representatives (including the lead compound designated C-1311) possess potent anti-tumour activity against a range of murine neoplasms (Cholody *et al.*, 1992; Kusnierczyk *et al.*, 1994) and human tumour xenografts in nude mice (Augustin *et al.*, 1996), in addition to significant *in vitro* cytotoxicity against both murine leukaemia cells (Cholody *et al.*, 1992) and the human tumour lines of the National Cancer Institute (NCI) screen. C-1311 has recently been accepted for clinical trials in the UK.

Recent *in vitro* studies have identified the imidazoacridinones as topoisomerase II poisons (Skladanowski *et al.*, 1996), and three major representatives (C-1311, C-1310, C-1263) have been shown to be potent inhibitors of L1210 (murine leukaemia) cell cycle progression, causing selective accumulation in the $G_2$ phase (i.e. $G_2$ block) (Augustin *et al.*, 1996). This cellular response is universally associated with the action of DNA-damaging agents, including the topoisomerase II inhibitors (Kimler *et al.*, 1978; Konopa, 1988; Lock and Ross, 1990*a*; O'Connor *et al.*, 1991; Sorenson and Eastman, 1988*b*; Tounekti *et al.*, 1993), and represents activation of a DNA damage cell cycle checkpoint, whose engagement delays segregation of damaged chromosomes, thereby preventing mitotic catastrophe, and affording the cell opportunity for repair (Al-Khodairy and Carr, 1992; Hartwell and Weinert, 1989; Murray, 1994; Murray, 1992; Weinert and Lydall, 1993). The biochemical basis of this cytostatic effect is relatively well understood, although the mechanism by which these agents actually effect cell killing, in which anti-tumour activity resides, remains comparatively obscure.

Reports of rapid or immediate cell death secondary to exposure to DNA-damaging agents (i.e. cell death without prior cycle arrest) have been made. However, these events are apparently restricted to cell types with a particular propensity for this response (Bertrand *et al.*, 1991*b*, 1993; Del Bino *et al.*, 1990; Kaufmann *et al.*, 1989; Radford *et al.*, 1994), or dependent upon the integrity of a p53-dependent programmed cell death pathway (Clarke *et al.*, 1993; Lowe *et al.*, 1993), which is itself only manifest in certain, mainly lymphoid, cell types (Fan *et al.*, 1994; O'Connor *et al.*, 1993*b*; Slichenmyer *et al.*, 1993).

The appearance of cells arrested in the $G_2$ phase has been shown to precede cell disintegration in many cell lines exposed to various DNA-damaging agents (Evans and Dive, 1993; Kim *et al.*, 1993; Kruman *et al.*, 1991; Lock and Ross, 1990*b*; Ormerod *et al.*, 1994; Skladanowski and Konopa, 1993; Sorenson and Eastman, 1988*a*; Warters, 1992; Yamagishi *et al.*, 1993), suggesting that cycle arrest is an essential intermediate in drug action. Consequently, considerable importance has been placed upon the role of events normally associated with the $G_2$/M transition in the death mechanism (Barry *et al.*, 1990; Bertrand *et al.*, 1991*a*; Eastman, 1990; Fotedar *et al.*, 1995; Rubin *et al.*, 1993; Shi *et al.*, 1994; Sorenson *et al.*, 1990), leading to the proposition that inappropriate or aberrant mitotic events may actually cause cell death.

The $G_2$/M transition is universally controlled in mammalian systems by $p34^{cdc2}$ kinase (Norbury and Nurse, 1992), the activity of which is principally regulated by association with cyclin B (Pines and Hunter, 1989) and a complex series of phosphorylation/dephosphorylation reactions (Draetta and Beach, 1988). Cyclin A is also implicated in association with both $p34^{cdc2}$ and one of a family of related protein kinases (Meyerson *et al.*, 1992), identified as $p33^{cdk2}$ (Elledge *et al.*, 1992; Rosenblatt *et al.*, 1992). Mitotic progression is ultimately achieved by final activation of the $p34^{cdc2}$/cyclin B complex through dephosphorylation of $p34^{cdc2}$ at threonine 14 and tyrosine 15, and it is inhibition of this tyrosine dephosphorylation (Lock, 1992; Lock and Keeling, 1993; O'Connor *et al.*, 1993*a*), with concomitant suppression of $p34^{cdc2}$ kinase activity (Lock and Ross, 1990*a*; O'Connor *et al.*, 1992; Tsao *et al.*, 1992), which effects the premitotic cell cycle arrest induced by DNA-damaging agents.

At the molecular level, the proposition that (aberrant) mitotic events cause cell death is amply supported by recent evidence. Activation (tyrosine dephosphorylation) of $p34^{cdc2}$ kinase at inappropriate times during the cell cycle leads to cell death (Shi *et al.*, 1994). Overexpression of cotransfected $p34^{cdc2}$ and cyclin B in BHK cells induces mitotic catastrophe

Correspondence: D Wheatley
Received 28 February 1996; revised 21 May 1996; accepted 29 May 1996

(Heald *et al.*, 1993) and comparable ectopic expression of this cyclin–kinase complex in HeLa cells results in DNA fragmentation and cell death, while cyclin B-specific antisense oligonucleotides can suppress the lethal transition (Fotedar *et al.*, 1995). Similarly, activation of $p34^{cdc2}$ and $p33^{cdk2}$ by methylxanthines and purine analogues induces cell death in arrested HeLa cells, the effect being accompanied by an increase in histone H1 kinase activity to near mitotic levels (Meikrantz *et al.*, 1994).

Indeed, the release of cells from DNA damage-induced $G_2$ arrest into cell death is accompanied by an increase in $p34^{cdc2}$ kinase activity (Lock and Keeling, 1993; Lock and Ross, 1990*b*) and is promoted or potentiated by the same agents known to cause premature mitosis, whose actions enhance $p34^{cdc2}$ tyrosine dephosphorylation (Crompton *et al.*, 1993; Hain *et al.*, 1993; Lock *et al.*, 1994; Steinmann *et al.*, 1991; Tam and Schlegel, 1992). It seems likely then that this particular mode of cell death: (1) is dependent upon assembly of the primed mitotic machinery, specifically the hyperphosphorylated $p34^{cdc2}$/cyclin B complex; and (2) reflects a failure of the $G_2$ checkpoint to maintain a premitotic arrest in a circumstance where cell cycle progression is incompatible with cellular survival.

That DNA damage-induced cell death is dependent upon the assembly of the hyperphosphorylated $p34^{cdc2}$/cyclin B complex associated with $G_2$ phase, has considerable significance and clinical implication. It is known that the range of drug concentrations causing 'pure' $G_2$ block is very narrow. An increase in levels beyond that producing $G_2$ accumulation additionally induces S-phase arrest (Konopa, 1988); a stage in cycle before the assembly of the mitotic machinery. We, therefore, set out to characterise the cell cycle perturbations induced by C-1311 across a wide concentration range, and then to examine the cellular responses and treatment outcomes subsequent to these different primary cycle perturbations.

Our results show that, as predicted, drug levels exceeding those which induce a $G_2$ arrest effectively inhibit cell killing, demonstrating that high dose does not necessarily correlate with increased cell death, while at the same time providing further evidence for the role of events normally associated with the $G_2$/M transition in the cell death process.

### Materials and methods

### *Drugs, reagents and chemicals*

C-1311 (synthesised at the Technical University of Gdansk, Poland) was stored at 4°C as a stock solution (50 $\mu g\ ml^{-1}$ in phosphate-buffered saline (PBS); filter sterilised). RPMI-1640 and Dulbecco's modified Eagle (DME, 4500 $mg\ l^{-1}$ glucose modification) media were obtained from Gibco/BRL (Life Technologies Ltd., Paisley, UK). Calf serum was from Advanced Protein Products Ltd. (Brierley Hill, UK) and benzylpenicillin from Britannia Pharmaceuticals Ltd. (Redhill, UK). Streptomycin sulphate, spermine tetrahydrochloride, tris(hydroxymethyl)aminomethane, trypsin, trypsin inhibitor, ribonuclease A, propidium iodide, 3-(4,5-dimethylthiazol-2-yl)-2,5-diphenyl tetrazolium bromide (MTT), and isopropanol were purchased from Sigma. Nonidet P40 and dimethyl sulphoxide (DMSO) were from BDH Chemicals Ltd. (Poole, UK), and crystal violet from Raymond A Lamb (London, UK).

### *Cell culture*

HeLa S3 (human cervical epithelial carcinoma) cells were cultured as a monolayer in RPMI-1640 supplemented with 10% calf serum and antibiotics (100 $\mu g\ ml^{-1}$ streptomycin, 100 $U\ ml^{-1}$ penicillin) at 37°C in a humidified 5% carbon dioxide atmosphere. Under these conditions, the population doubling time was approximately 24 h. Primary human diploid fibroblasts (RMF/), derived from neonatal foreskin samples, were maintained in monolayer in DME supplemented with 10% calf serum and antibiotics as above. Cells from passages 6 to 10 were used in these studies. Cells were provided by BioCuRe Ltd. (Aberdeen, UK).

### *Drug treatments*

A series of C-1311 working solutions were prepared from stock, with PBS as diluent, immediately before use. Samples (100 $\mu l$) of each, added directly to a culture (or to 2 ml of culture medium), achieved the specified final concentrations. Control cultures received an equivalent solvent exposure. Cells were grown in 6-well culture plates from a seeding density of $\sim 1\times10^5$ (HeLa) or $\sim 5\times10^4$ (RMF/) cells per well (in 2 ml of culture medium) for 24 h before drug addition. Cultures were dosed as described. After drug exposure, cultures were either submitted for analysis or the culture medium aspirated, the monolayer washed with prewarmed PBS and incubation continued in fresh medium for up to a total of 96 h, at which point the same analyses were conducted.

### *Flow cytometry*

Cells were harvested by gentle trypsinisation and combined with any non-adherent cells contained within the aspirated medium, which also served to neutralise the trypsin action. The cells were collected by centrifugation (400 *g*, 5 min) and resuspended in PBS. An aliquot of this suspension was further diluted with PBS and submitted for cell sizing; 5000 cells were analysed. Nuclei from the remainder of the suspension were isolated and stained with propidium iodide by the method of Vindeløv *et al.* (1983). Nuclear fluorescence (DNA content) and forward light scatter (cell or nuclear volume) were recorded for each population, with doublet discrimination, using an EPICS Profile-II flow cytometer (Coulter Electronics Inc., Hialeah, FL, USA). The number of nuclei present in each (constant volume) sample was counted (concurrent with fluorescence and light scatter recordings) to give an indication of the cell density in each culture. Not less than 10 000 nuclei were processed per sample. Cell cycle phase distributions and the number of sub-$G_1$ events (expressed as a percentage of total events) were obtained, as necessary, by decomposition of single parameter DNA content frequency histograms using the 'Cytologic' software package (Coulter Electronics Inc.).

### *Growth inhibition assay*

Cells were grown in 6-well culture plates for 24 h from a seeding density of $\sim 5\times10^4$ cells per well. Cultures were dosed as described and incubation continued for a further 72 h, at which point the culture medium was removed, the monolayers washed with prewarmed PBS, and the cells harvested by gentle trypsinisation. Cell number was determined with a Model ZM Coulter Counter (Coulter Electronics Inc.) and the percentage of growth inhibition was calculated as follows, after the method of Bhuyan *et al.*, (1992): 100 − 100 × [(cell number in treated well − cells inoculated) ÷ (cell number in control well − cells inoculated)]. The 50% growth inhibitory concentration ($IC_{50}$) was determined by interpolation of the resultant dose–response curves.

### *Clonogenic survival assay*

Reproductive capacity was assessed by colony-forming assay. Cells in exponential growth were washed twice with prewarmed PBS and resuspended in growth medium. Cell density was determined and adjusted such that a known number ($\sim$1000 cells) were seeded into each well of a 6-well culture plate in 2 ml of medium. Cells were allowed to grow for 24 h before being dosed as described. After 3 h drug exposure, the medium was removed and the cells washed with prewarmed drug-free medium. Fresh medium was added and incubation continued for a further 5 days. Colonies were stained with 2% crystal violet in methanol and counted. Reproductive capacity was expressed as a percentage of the cloning efficiency of controls.

### *MTT cleavage assay*

Cell survival was assessed by MTT cleavage assay (Mosmann, 1983). Cells were seeded in 96-well culture plates at ~4000 cells per well in 100 $\mu$l of growth medium and incubated at 37°C for 24 h, at which time the medium was removed and replaced by complete medium (100 $\mu$l) containing C-1311. After 3 h, the medium was aspirated and the monolayers washed with prewarmed PBS. Fresh medium (100 $\mu$l) was added to each well and incubation continued for a further 93 h. MTT solution (10 $\mu$l) (5 mg ml$^{-1}$ in PBS, filter sterilised) was added to each well and incubation continued for a further 4 h at 37°C. The formazan product was solubilised by the addition of 100 $\mu$l of 0.04 M hydrochloric acid in isopropanol. The optical density of each well was measured using a Dynatech MR5000 plate reader at a wavelength of 570 nm with background subtraction at 690 nm. Wells containing culture medium and MTT but no cells acted as blanks. Cell survival was expressed as a percentage defined by: [(drug − blank) ÷ (control − blank) x 100].

### Results

### *Growth inhibition and cell cycle perturbations following continuous exposure*

Dose-dependent inhibition of HeLa S3 cell growth was observed after 72 h continuous exposure to C-1311 (Figures 1 and 2). The $IC_{50}$ was calculated as $0.018 \pm 0.003$ $\mu$g ml$^{-1}$ ($n = 4$). This value is in broad agreement with the work of Cholody *et al.* (1992) and Augustin *et al.* (1996), and indicates that HeLa and L1210 murine leukaemia cells share a similar sensitivity to the growth-inhibitory activity of the compound.

**Figure 1** Structural formula of the substituted aminoimidazoacridinone designated C-1311.

**Figure 2** The growth-inhibitory effect of 72h continuous treatment with C-1311 on HeLa S3 cells. Each dose–response curve represents an independent experiment.

The influence of C-1311 on the cell cycle progression of exponentially growing asynchronous cultures of HeLa cells was assessed after continuous exposure to a wide range of drug concentrations. Histograms representing the distribution of cells through the cycle after a 24 h exposure are shown as Figure 3a and the corresponding cell cycle phase distributions are presented as Table I.

Cultures treated with C-1311 at 0.01 $\mu$g ml$^{-1}$ showed only a marginal deviation from control cell cycle distribution. Exposure to C-1311 at 0.05 $\mu$g ml$^{-1}$ induced a conspicuous perturbation of cell cycle progression, however. A marked increase in the proportion of cells with a $G_2$ phase character, and a significant reduction in cell number over control was observed, defining a $G_2$ phase accumulation. Given that exposure time was selected to be marginally greater than cycle time, it was supposed that the observed $G_1$ population was derived from cells having recently made the $G_2$/M transition, and having passed the $G_2$ accumulation point, signifying either that $G_2$ transit time was extended, the progression delay was of a finite duration, or that some cells did not arrest at the accumulation point (i.e. an incomplete or 'leaky' cell cycle arrest). No slowing of S-phase transit was apparent. Treatment at 0.1 $\mu$g ml$^{-1}$ yielded a culture composed almost entirely of cells with $G_2$ phase character. This, together with the albeit total absence of cells from the $G_1$ and S-phases indicated a complete and preferential $G_2$ arrest. Elevation of drug levels beyond this concentration resulted in the induction of an additional S-phase accumulation, with increasing concentration producing accumulations earlier in phase.

Exponentially growing asynchronous cultures of normal human diploid fibroblasts were continuously exposed to C-1311 for 48 h. The agent induced the same sequence of cycle perturbations, and at similar concentrations, as in HeLa cells, although in all cases a $G_1$ phase arrest accompanied the late cycle accumulations (Figure 3b; Table II). Nuclear and cellular volume distributions were consistent with the reported perturbations of cell cycle (data not shown).

### *Treatment outcomes following acute exposures*

Exponentially growing HeLa S3 cultures were exposed to C-1311 at concentrations of 0.01 – 1.0 $\mu$g ml$^{-1}$ for 3 h, before transfer into drug-free medium for up to a total of 96 h. Histograms of nuclear DNA content obtained throughout this treatment matrix are shown as Figure 4.

While untreated cultures exhibited typically unperturbed DNA content distributions throughout the course of the experiment, treatment with C-1311 at 0.01, 0.05, or 0.1 $\mu$g ml$^{-1}$ induced modest late cycle phase accumulations, which were maximal at 12 h. These cultures recovered control cell cycle distributions within the time course of the experiment and cell number increased correspondingly. These cultures were subject to a transient or incomplete $G_2$ block, which was rapidly overcome, thus representing only a minor impediment to expansion of the culture. Exposure to C-1311 at 0.5 $\mu$g ml$^{-1}$ induced an overt and progressive disturbance of cell cycle distribution, however. Almost complete absence of cells with a $G_1$ character at 12 h signified a strict $G_2$ block, confirmed by an all but 'pure' $G_2$ population at 24 h. By 48 h, this arrested population was accompanied by considerable nuclear debris (sub-$G_1$ signal) signifying extensive degradation of the culture. All cells arrested in $G_2$ phase at 48 h were subsequently lost, such that by 96 h only nuclear debris remained. Treatment at a concentration of 1.0 $\mu$g ml$^{-1}$ also produced frank perturbations of cell cycle distribution, but with a markedly different outcome. In contrast to treatment at 0.5 $\mu$g ml$^{-1}$, these cultures were composed predominantly of S-phase cells at 24 h and, although these cells attained approximate $G_2$ DNA content character within a further 24 h, the absence of nuclear debris at this time was striking. Indeed, by the end of the experiment (96 h), there was only limited evidence of cellular disintegration. Cell and nuclear volume distribu-

**Figure 3** Histograms representing the distribution of **(a)** HeLa S3 cells and **(b)** normal human diploid fibroblasts (RMF/) through the cell cycle following continuous treatment with C-1311 for 24 h or 48 h respectively. Each histogram is a plot of nuclear DNA content against frequency. ↑ represents 2n DNA content, ⇑ represents 4n DNA content. These data were obtained from a single experiment but are representative of **(a)** five and **(b)** three independent experiments.

**Table I** Cell cycle phase distributions of HeLa cells treated continuously for 24 h with various concentrations of C-1311. These cell cycle phase fractions were derived from the DNA content frequency histograms shown in Figure 3a

| *C-1311 concentration ($\mu g\,ml^{-1}$)* | *Percentage of cells* $G_1$ | *S* | $G_2/M$ |
|---|---|---|---|
| Control | 58 | 27 | 15 |
| 0.01 | 49 | 27 | 23 |
| 0.05 | 28 | 11 | 61 |
| 0.1 | 3 | 0 | 97 |
| 0.5 | 2 | 60 | 38 |
| 1.0 | 3 | 57 | 40 |

**Table II** Cell cycle phase distributions of normal human diploid fibrobalsts (RMF/) treated continuously for 48 h with various concentrations of C-1311. These cell cycle fractions were derived from the DNA content frequency histograms shown in figure 3b.

| *C-1311 concentration ($\mu g\,ml^{-1}$)* | *Percentage of cells* $G_1$ | *S* | $G_2/M$ |
|---|---|---|---|
| Control | 75 | 12 | 13 |
| 0.05 | 66 | 0 | 34 |
| 0.1 | 63 | 3 | 34 |
| 0.25 | 62 | 8 | 29 |
| 0.5 | 60 | 11 | 29 |
| 1.0 | 59 | 17 | 24 |

tions, and microscopic examinations were consistent with these findings (data not shown). Cell death superficially resembled apoptosis (appearance of small, rounded, refractile bodies) but a panel of techniques for identification of the features of this form of cell death together failed to provide unequivocal confirmation. On this point we concur with the sentiments of Darzynkiewicz (1995) that cell death from the $G_2$ compartment often resembles apoptosis, although it frequently lacks all the classical features of the latter.

Reproductive capacity and cellular survival at 96 h, as assessed by colony-forming ability and MTT cleavage following the same drug exposures, were examined in parallel with this flow cytometric analysis. Reproductive capacity was reduced in a dose-dependent manner, with no cells forming colonies after treatment at drug concentrations of 0.5 $\mu g\,ml^{-1}$ and above (Figure 5). In contrast, cell survival was reduced in a dose-dependent fashion to a minimum at 0.5 $\mu g\,ml^{-1}$ but, beyond this level, the typical sigmoidal profile was inflected, and survival was significantly enhanced from this minimum (Figure 6).

A further series of flow cytometric experiments were conducted, in which exposure to C-1311 was extended to 6, 12 and 24 h before transfer to drug-free medium. Essentially the same sequence of events was mirrored in these circumstances, although at these extended exposure times, cellular disintegration was apparent at lower drug levels and became manifest more rapidly. Cellular persistence was also seen at correspondingly lower drug levels. The dual parameters of dose and exposure time were important in

**Figure 4** Histograms of nuclear DNA content for HeLa S3 cells acutely treated with C-1311. Following drug exposure (0–1.0 $\mu g\,ml^{-1}$ for 3 h), cells were incubated in drug-free medium for the indicated intervals up to a total of 96 h. Number of sub-$G_1$ events (expressed as a percentage of total) for each histogram is given where > 3%. Details as Figure 3. Each treatment was repeated at least once.

**Figure 5** Reproductive capacity of HeLa S3 cells exposed to C-1311 for 3 h as assessed by colony-forming assay. Cloning efficiency of untreated (control) cells was 25–30%. Each point represents the mean of two independent determinations.

**Figure 6** Survival of HeLa S3 cells exposed to C-1311 for 3 h before transfer to drug-free medium to a total of 96 h. Cell survival was assessed by MTT cleavage assay. Each point represents the mean of six replicate determinations ± standard deviation. Control $OD_{570-690}$ was 0.985 ± 0.031. These data were obtained from a single experiment but are representative of three independent experiments.

determining the treatment outcome. In demonstration of this effect, Figure 7 presents photomicrographs of HeLa cultures exposed to C-1311 over a concentration range from 0.01 to 1.0 $\mu$g ml$^{-1}$ for 24 h before transfer into drug-free medium for only a further 24 h. Considerable detachment of cells from the monolayer, which we associate with cell death, was apparent in cultures dosed at 0.05 $\mu$g ml$^{-1}$ and extensive at 0.1 $\mu$g ml$^{-1}$ (Figure 7c and d). Cultures exposed to greater drug concentrations (0.5 and 1.0 $\mu$g ml$^{-1}$), although composed of apparently enlarged and misshapen cells, exhibited no 'rounded' or detached bodies and appeared relatively healthy (Figure 7e and f). We note with interest that cultures dosed at these higher levels remained largely free of cell disintegration even after a further 72 h in drug-free medium (data not shown).

### Discussion

We have studied the actions of C-1311, the lead compound in a new class of imidazoacridinone antineoplastic agents, against a human tumour line (HeLa S3), in an examination of the events associated with the lethality of these agents.

First, we examined the sequence of dose-dependent cell cycle effects induced by C-1311 and found them to be typical of a DNA-damaging agent and consistent with the established pattern of cell cycle checkpoint engagement. We demonstrated that C-1311 can induce a complete preferential 'pure' $G_2$ accumulation (*i.e.* $G_2$ arrest) in HeLa cultures, representing the action of a DNA damage cell cycle checkpoint, but that the concentration range of this effect is very narrow, with elevation of the drug level inducing

**Figure 7** Phase contrast photomicrographs of HeLa S3 cultures exposed to C-1311 at **(a)** control, **(b)** 0.01, **(c)** 0.05, **(d)** 0.1, **(e)** 0.5 and **(f)** 1.0 $\mu$g ml$^{-1}$ for 24 h before transfer to drug-free medium for a further 24 h. Bar = 50 $\mu$m.

additional and increasing S-phase accumulation (Konopa, 1988), more likely caused by a physical impediment to genomic replication than to an active response mechanism (Fornace, 1992; Liu, 1989). This sequence of events is similar to, and induced at similar drug levels as, those observed against the L1210 murine leukaemia line (Augustin *et al.*, 1996), and is, therefore, consistent with our finding that the sensitivity of the L1210 line to (the growth inhibitory activity of) the compound reported by others (Cholody *et al.*, 1992) is shared by the HeLa line.

We have also shown that normal human diploid fibroblasts (which putatively express wild-type p53 tumour-suppressor gene) exposed to C-1311 exhibit the same sequence of cycle perturbations, and at similar concentrations as HeLa cells, although in all cases a $G_1$ phase arrest accompanied these late cycle accumulations. This observation is consistent with the demonstration that cells which express wild-type p53 exhibit both $G_1$ and $G_2$ arrest after exposure to DNA-damaging agents, whereas those which lack p53 expression, express a mutant form of the p53 gene, or fail to accumulate p53 protein (as do HeLa cells), typical of many tumour cell lines (Hollstein *et al.*, 1991; Levine *et al.*, 1991), arrest only in $G_2$, reflecting loss of the $G_1$ DNA damage-sensitive cell cycle checkpoint (Fritsche *et al.*, 1993; Kastan *et al.*, 1991, 1992; Kuerbitz *et al.*, 1992).

An identical sequence of cycle perturbations was exhibited by cultures of HeLa cells and normal fibroblasts treated with the recognised topoisomerase II inhibitor, etoposide (Liu, 1989), albeit at 10-fold higher concentrations than with C-1311 (unpublished observations).

Cell cycle arrest is not a biological end point, however, and it is the fate of the arrested cell which determines the ultimate treatment outcome. Flow cytometric analysis of cellular DNA content can reveal both cell cycle position and, as judged by the appearance of cells with a less than $G_1$ DNA content, nuclear disintegration. While quantitation of sub-$G_1$ events does not give an absolute measure of cell death, since more than one recordable fragment may result from disintegration of a single cell, this technique does provide a rapid method for the simultaneous analysis of the kinetics of cell cycle perturbation and cell death. In this way we demonstrated that HeLa cultures acutely exposed to doses of C-1311 that induce a preferential and persistent $G_2$ arrest, subsequently undergo cell death from this compartment. This is in accordance with numerous empirical reports, which identify arrest in this phase as an immediate precursor to cell death induced by chemotherapeutic DNA-damaging agents (see Introduction for references). Additionally, however, doses exceeding the narrow range in which 'pure' $G_2$ accumulations were generated, paradoxically, did not result in the same extensive cell death. Instead these cultures, initially composed predominantly of S-phase cells, remained relatively healthy, showing comparatively little of the cellular disintegration and death so striking in their cohort cultures.

Cellular survival assessments made by MTT cleavage assay, which relies upon the ability of live (but not necessarily proliferating) cells to convert a soluble component to an insoluble and readily quantifiable coloured product through the action of mitochondrial dehydrogenase (Mosmann, 1983), clearly reflected the enhanced survival of cultures exposed to high doses of compound, relative to treatments at lower levels, as did two other independent metabolic measures: neutral red uptake and [$^3$H]leucine incorporation (data not shown). Clonogenic assays cannot discriminate between arrested cells which do not form colonies and those which are killed outright (Lanks and Lehman, 1990); taken together, these observations identify the surviving population as non-reproductive, but metabolically active.

We consider our findings entirely consistent with the proposition that events normally associated with the $G_2$/M transition are implicated in the mechanisms of cell death, and indeed provide further evidence in its support. That cell death is associated with activation (dephosphorylation) of the $p34^{cdc2}$ kinase distal to $G_2$ arrest (Lock *et al.*, 1994; Lock and Keeling, 1993; Lock and Ross, 1990*b*), suggests that this particular mode of cell killing is dependent upon assembly of the primed mitotic machinery, specifically the hyperphosphorylated $p34^{cdc2}$/cyclin B complex. This mechanism relies, therefore, upon cell cycle progression being allowed to proceed to the temporal location of complex assembly. Given that human cells synthesise cyclin B and form $p34^{cdc2}$ complexes comparatively late in cycle (Steinmann *et al.*, 1991), cellular derangements which prevent progression to this point, such as the impediment to genomic replication owing to high doses of DNA-damaging agents observed in this study and by others (Konopa, 1988), will prevent assembly of the complex. Although cellular damage secondary to pharmacological insult may be more severe, cell death induced by kinase activation is prevented, just as by artificial inhibition of complex assembly by cyclin B antisense oligonucleotides (Fotedar *et al.*, 1995).

Cells treated with high drug levels, which initially exhibited an S-phase delay, and appeared protected from cell death, did eventually attain $G_2$ DNA content but still did not succumb. Instead we found a tendency to initiate a second round of DNA synthesis in the absence of mitosis (see $>G_2$ cells Figure 4, 1.0 $\mu$g ml$^{-1}$ at 48 h and 96 h), similar to behaviour exhibited by cells surviving doxorubicin exposure (Lanks and Lehman, 1990). It is reported that HeLa cells subject to inhibition of DNA synthesis also down-regulate protein synthesis due to a strict integrational coupling between nuclear and cytoplasmic events, and hence do not accumulate cyclin B when so arrested (Kung *et al.*, 1993). It may be that excessive delay in genomic replication is associated with an inability to accumulate sufficient levels of the protein for mitosis (or cell death), resulting in survival and entry to the higher ploidy state observed. Studies are underway to confirm directly the failure of these cells to accumulate or associate cyclin B and $p34^{cdc2}$.

Application of our findings to the clinical exploitation of DNA-damaging chemotherapeutic agents suggests that maximum anti-tumour efficacy can only be attained within a narrow dose range, and that doses in excess of this level may not confer any advantage in terms of tumour cell kill. Cells surviving a DNA damage insult, which presumably harbour a pool of induced genetic mutations, can regain proliferative capacity (Sorensen and Eastman, 1988*b*) but may also become 'adapted' to their lesions and resume cell cycle progression before completion of repair (Weinert and Lydall, 1993). The treatment thus becomes associated with an enhanced probability that more malignant or drug-resistant clones will appear, and hence with a poorer outcome. Only those treatments which culminate in frank tumour cell death are compatible with the pursuit of selective antineoplastic chemotherapy.

It is still unclear whether cell death associated with mitotic kinase activation results directly from failure to execute a normal mitosis, and whether in some circumstances this mitotic catastrophe merely resembles programmed cell death (*i.e.* by causing the features of apoptosis), or if proteins phosphorylated by $p34^{cdc2}$/cyclin B are positive mediators of a true, specific, inducible cell death programme. The latter is predicted by a modification of the 'dual signal' hypothesis (Evan and Littlewood, 1993), in which progressional control events simultaneously activate both the proliferative process and a cell death mechanism as a 'fail safe' against uncontrolled or inappropriate proliferation. Relaxation of the normal downstream inhibition of the cell death programme, on detection of unrepaired genomic damage, provides a physiological suicide response after checkpoint failure rather than death as a consequence of mitosis attempted in the presence of critical damage with the attendant danger of survival with gross chromosomal derangement.

What is clear, however, is that this pathway frequently constitutes the predominant route to the death of tumour cells, at least *in vitro*, following induced DNA damage. Whatever the detail, downstream failure of the premitotic checkpoint – leading to inappropriate or premature kinase activation – is an important initial feature of this mechanism. The 'proliferative impetus' gained in transformation through overexpression of the positive regulators of cell cycle control, p34[cdc2] and cyclin B (Oshima *et al.*, 1993; Steinmann *et al.*, 1994), which shift the balance in regulation away from the antiproliferative signals derived from DNA damage, with concomitant attenuation of $G_2$ checkpoint function (Kaufmann *et al.*, 1995), facilitates the genetic instability characteristic of cancer (Hartwell and Kastan, 1994; Hartwell, 1992; Weinert and Lydall, 1993), but also predisposes these cells with a vulnerability to DNA damage. Given, additionally, that checkpoint function appears inversely related to increasing transformed phenotype (Kung *et al.*, 1990), recent indications that cells with defective p53 are more susceptible to $G_2$ checkpoint abrogation (Fan *et al.*, 1995; Powell *et al.*, 1995; Russell *et al.*, 1995), and the marked susceptibility of the highly deranged HeLa line (this study), future work will attempt to correlate $G_2$ checkpoint stringency with sensitivity to DNA-damaging agents to examine possible differential lethality towards tumour cells.

It has recently been demonstrated that $G_1$ arrest mediated by superimposition of wild-type p53 function upon a transformed cell line markedly reduced the extent of DNA damage-induced cell death, coincident with a decrease in the proportion of cells reaching the $G_2$ phase (Malcomson *et al.*, 1995), and that abolition of $G_1$ arrest through disruption of normal p53 function sensitised a human cancer line to DNA damage, coincident with an increase in the proportion of cells reaching $G_2$ (Fan *et al.*, 1995). These findings further highlight the importance of $G_2$ checkpoint targeting for maximum chemotherapeutic advantage, especially in cell types which do not exhibit a propensity to undergo p53-dependent programmed cell death. On this basis, the influence of $G_1$ checkpoint fidelity upon the prevalence of cell killing from the $G_2$ compartment will also be examined in terms of both chemoresistance of tumour cells, and protection of normal cells, like the fibroblasts in this study, from deleterious consequences of exposure to these noxious agents.

**Acknowledgements**
This work has been made possible by the generous gift of C-1311 from Professor Jerzy Konopa (Department of Pharmaceutical Technology and Biochemistry, Technical University of Gdansk, Poland), the advice of Dr Ewa Augustin (of the same institution), and grants from The Cancer Endowments Fund of the University of Aberdeen. JL is a Caledonian Research Foundation Scholar.

### References

AL-KHODAIRY F AND CARR AM. (1992). DNA repair mutants defining the G2 checkpoint pathways in *Schizosaccharomyces pombe*. *EMBO J.*, **11**, 1343–1350.

AUGUSTIN E, WHEATLEY DN, LAMB J AND KONOPA J. (1996). Imidazoacridinones arrest cell cycle progression in G2 phase of L1210 cells. *Cancer Chemother. Pharmacol.*, **38**, 39–44.

BARRY MA, BEHNKE CA AND EASTMAN A. (1990). Activation of programmed cell death (apoptosis) by cisplatin, other anticancer drugs, toxins and hyperthermia. *Biochem. Pharmacol.*, **40**, 2353–2362.

BERTRAND R, KERRIGAN D, SARANG M AND POMMIER Y. (1991*a*). Cell death induced by topoisomerase inhibitors. *Biochem. Pharmacol.*, **42**, 77–85.

BERTRAND R, SARANG M, JENKIN J, KERRIGAN D AND POMMIER Y. (1991*b*). Differential induction of secondary DNA fragmentation by topoisomerase II inhibitors in human tumour cell lines with amplified c-*myc* expression. *Cancer Res.*, **51**, 6280–6285.

BERTRAND R, SOLARY E, JENKINS J AND POMMIER Y. (1993). Apoptosis and its modulation in human promyelocytic HL-60 cells treated with DNA topoisomerase I and II inhibitors. *Exp. Cell. Res.*, **207**, 388–397.

BHUYAN BK, SMITH KS, ADAMS EG, PETZOLD GL AND MCGOVREN JP. (1992). Lethality, DNA alkylation, and cell cycle effects of Adozelesin (U-73975) on rodent and human cells. *Cancer Res.*, **52**, 5687–5692.

CHOLODY WM, MARTELLI S AND KONOPA J. (1992). Chromophore-modified antineoplastic imidazoacridinones. Synthesis and activity against murine leukemias. *J. Med. Chem.*, **35**, 378–382.

CHU G. (1994). Cellular responses to cisplatin. *J. Biol. Chem.*, **269**, 787–790.

CLARKE AR, PURDIE CA, HARRISON DJ, MORRIS RG, BIRD CC, HOOPER ML AND WYLLIE AH. (1993). Thymocyte apoptosis induced by p53-dependent and independent pathways. *Nature*, **362**, 849–852.

CROMPTON NEA, HAIN J, JAUSSI R AND BURKART W. (1993). Staurosporine- and radiation-induced G2-phase cell cycle blocks are equally released by caffeine. *Radiat. Res.*, **135**, 372–379.

DARZYNKIEWICZ Z. (1995). Apoptosis in antitumour strategies: modulation of cell cycle or differentiation. *J. Cell. Biochem.*, **58**, 151–159.

DEL BINO G, SKIERSKI JS AND DARZYNKIEWICZ Z. (1990). Diverse effects of camptothecin, an inhibitor of topoisomerase I, on the cell cycle of lymphocytic (L1210, MOLT-4) and myelogenous (HL-60, KG1) leukemic cells. *Cancer Res.*, **50**, 5746–5750.

DRAETTA G AND BEACH D. (1988) Activation of cdc2 protein kinase during mitosis in human cells: cell cycle-dependent phosphorylation and subunit rearrangement. *Cell.*, **54**, 17–26.

EASTMAN A. (1990). Activation of programmed cell death by anticancer agents: cisplatin as a model system. *Cancer Cells.*, **2**, 275–280.

ELLEDGE SJ, RICHMAN R, HALL FL, WILLIAMS RT, LODGSON N AND HARPER JW. (1992). CDK2 encodes a 33-kDa cyclin A-associated protein kinase and is expressed before CDC2 in the cell cycle. *Proc. Natl Acad. Sci. USA*, **89**, 2907–2911.

EVAN GI AND LITTLEWOOD TD. (1993). The role of c-*myc* in cell growth. *Curr. Opin. Genet. Dev.*, **3**, 44–49.

EVANS DL AND DIVE C. (1993). Effects of cisplatin on the induction of apoptosis in proliferating hepatoma cells and nonproliferating immature thymocytes. *Cancer Res.*, **53**, 2133–2139.

FAN S, EL-DEIRY WS, BAE I, FREEMAN J, JONDLE D, BHATIA K, FORNACE AJ, MAGRATH I, KOHN KW AND O'CONNOR PM. (1994). p53 gene mutations are associated with decreased sensitivity of human lymphoma cells to DNA damaging agents. *Cancer Res.*, **54**, 5824–5830.

FAN S, SMITH ML, RIVET DJ, DUBA D, ZHAN Q, KOHN KW, FORNACE AJ AND O'CONNOR PM. (1995). Disruption of p53 function sensitizes breast cancer MCF-7 cells to cisplatin and pentoxifylline. *Cancer Res.*, **55**, 1649–1654.

FORNACE AJ. (1992). Mammalian genes induced by radiation: activation of genes associated with growth control. *Annu. Rev. Genet.*, **26**, 507–526.

FOTEDAR R, FLATT J, GUPTA S, MARGOLIS RL, FITZGERALD P, MESSIER H AND FOTEDAR A. (1995). Activation-induced T-cell death is cell cycle dependent and regulated by cyclin B. *Mol. Cell. Biol.*, **15**, 932–942.

FRITSCHE M, HAESSLER C AND BRANDNER G. (1993). Induction of nuclear accumulation of the tumor-suppressor protein p53 by DNA-damaging agents. *Oncogene*, **8**, 307–318.

HAIN J, CROMPTON NEA, BURKART W AND JAUSSI R. (1993). Caffeine release of radiation induced S and G2 phase arrest in V79 hamster cells: increase of histone messenger RNA levels and p34[cdc2] activation. *Cancer Res.*, **53**, 1507–1510.

HARTWELL LH. (1992). Defects in a cell cycle checkpoint may be responsible for the genomic instability of cancer cells. *Cell*, **71**, 543–546.

HARTWELL LH AND KASTAN MB. (1994) Cell cycle control and cancer. *Science*, **266**, 1821–1828.

HARTWELL LH AND WEINERT TA. (1989). Checkpoints: controls that ensure the order of cell cycle events. *Science*, **246**, 629–634.

HEALD R, MCLOUGHLIN M AND MCKEON F. (1993). Human wee1 maintains mitotic timing by protecting the nucleus from cytoplasmically activated cdc2 kinase. *Cell*, **74**, 463–474.

HOLLSTEIN M, SIDRANSKY D, VOGELSTEIN B AND HARRIS CC. (1991). p53 mutations in human cancers. *Science*, **253**, 49–53.

KASTAN MB, ONYEKWERE O, SIDRANSKY D, VOGELSTEIN B AND CRAIG RW. (1991). Participation of p53 protein in the cellular response to DNA damage. *Cancer Res.*, **51**, 6304-6311.

KASTAN MB, ZHAN Q, EL-DEIRY WS, CARRIER F, JACKS T, WALSH WV, PLUNKETT BS, VOGELSTEIN B AND FORNACE AJ. (1992). A mammalian cell cycle checkpoint pathway utilizing p53 and GADD45 is defective in ataxia-telangiectasia. *Cell*, **71**, 587-597.

KAUFMANN SH. (1989). Induction of endonucleolytic DNA cleavage in human acute myelogenous leukemia cells by etoposide, camptothecin, and other cytotoxic anticancer drugs: a cautionary note. *Cancer Res.*, **49**, 5870-5878.

KAUFMANN SK, LEVEDAKOU EB, GRADY HL, PAULES RS AND STEIN GH. (1995). Attenuation of G2 checkpoint function precedes human cell immortalization. *Cancer Res.*, **55**, 7-11.

KIM I-K, LEE J-H, SOHN H-S AND KIM S-H. (1993). Prostaglandin A2 and $\Delta^{12}$-prostaglandin J2 induce apoptosis in L1210 cells. *FEBS Lett.*, **321**, 209-214.

KIMLER BF, SCHNEIDERMAN MH AND LEEPER DB. (1978). Induction of concentration-dependent blockade in the G2 phase of the cell cycle by cancer chemotherapeutic agents. *Cancer Res.*, **38**, 809-814.

KONOPA J. (1988). G2 block induced by DNA crosslinking agents and its possible consequences. *Biochem. Pharmacol.*, **37**, 2303-2309.

KRUMAN II, MATYLEVICH NP, BELETSKY IP, AFANASYEV VN AND UMANSKY SR. (1991). Apoptosis of murine BW 5147 thymoma cells induced by dexamethasone and $\gamma$-irradiation. *J. Cell. Physiol.*, **148**, 267-273.

KUERBITZ SJ, PLUNKETT BS, WALSH WV AND KASTAN MB. (1992). Wild-type p53 is a cell cycle checkpoint determinant following irradiation. *Proc. Natl Acad. Sci. USA*, **89**, 7491-7495.

KUNG AL, SHERWOOD SW AND SCHIMKE RT.(1990). Cell line-specific differences in the control of cell cycle progression in the absence of mitosis. *Proc. Natl Acad. Sci. USA*, **87**, 9553-9557.

KUNG AL, SHERWOOD SW AND SCHIMKE RT.(1993). Differences in the regulation of protein synthesis, cyclin B accumulation, and cellular growth in response to the inhibition of DNA synthesis in Chinese hamster ovary and HeLa S3 cells. *J. Biol. Chem.*, **268**, 23072-23080.

KUSNIERCZYK H, CHOLODY WM, PARADZIEJ-LUKOWICZ J, RADZIKOWSKI C AND KONOPA J. (1994). Experimental antitumor activity and toxicity of the selected triazolo- and imidazoacridinones. *Arch. Immunol. Ther. Exp.*, **42**, 415-423.

LANKS KW AND LEHMAN JM. (1990). DNA synthesis by L929 cells following doxorubicin exposure. *Cancer Res.*, **50**, 4776-4778.

LEVINE AJ, MOMAND J AND FINLAY CA. (1991). The p53 tumour suppressor gene. *Nature*, **351**, 453-456.

LIU LF. (1989). DNA topoisomerase poisons as antitumor drugs. *Annu. Rev. Biochem.*, **58**, 351-375.

LOCK RB. (1992). Inhibition of $p34^{cdc2}$ kinase activation, $p34^{cdc2}$ tyrosine dephosphorylation, and mitotic progression in Chinese hamster ovary cells exposed to etoposide. *Cancer Res.*, **52**, 1817-1822.

LOCK RB AND KEELING PK. (1993). Responses of HeLa and Chinese hamster ovary $p34^{cdc2}$/cyclin B kinase in relation to cell cycle perturbations induced by etoposide. *Int. J. Oncol.*, **3**, 33-42.

LOCK RB AND ROSS WE. (1990*a*). Inhibition of $p34^{cdc2}$ kinase activity by etoposide or irradiation as a mechanism of G2 arrest in Chinese hamster ovary cells. *Cancer Res.*, **50**, 3761-3766.

LOCK RB AND ROSS WE. (1990*b*). Possible role for $p34^{cdc2}$ kinase in etoposide-induced cell death of Chinese hamster ovary cells. *Cancer Res.*, **50**, 3767-3771.

LOCK RB, GALPERINA OV, FELDHOFF RC AND RHODES LJ. (1994). Concentration-dependent differences in the mechanisms by which caffeine potentiates etoposide cytotoxicity in HeLa cells. *Cancer Res.*, **54**, 4933-4939.

LOWE SW, SCHMITT EM, SMITH SW, OSBOURNE BA AND JACKS T. (1993). p53 is required for radiation-induced apoptosis in mouse thymocytes. *Nature*, **362**, 847-849.

MALCOMSON RDG, OREN M, WYLLIE AH AND HARRISON DJ. (1995). p53-independent death and p53-induced protection against apoptosis in fibroblasts treated with chemotherapeutic drugs. *Br. J. Cancer*, **72**, 952-957.

MEIKRANTZ W, GISSELBRECHT S, TAM SW AND SCHLEGEL R. (1994). Activation of cyclin A-dependent kinases during apoptosis. *Proc. Natl Acad. Sci. USA*, **91**, 3754-3758.

MEYERSON M, ENDERS GH, WU C-L, SU L-K, GORKA C, NELSON C, HARLOW E AND TSAI L-H. (1992). A family of human cdc2-related protein kinases, *EMBO J.*, **11**, 2909-2917.

MOSMANN T. (1983). Rapid colorimetric assay for cellular growth and survival: Application to proliferation and cytotoxicity assays. *J. Immunol. Methods*, **65**, 55-63.

MURRAY AW. (1994). Cell cycle checkpoints. *Curr. Opin. Cell Biol.*, **6**, 872-876.

MURRAY AW. (1992). Creative blocks: cell-cycle checkpoints and feedback controls. *Nature*, **359**, 599-604.

NORBURY C AND NURSE P. (1992). Animal cell cycles and their control. *Annu. Rev. Biochem.*, **61**, 441-470.

O'CONNOR PM, WASSERMANN K, SARANG M, MAGRATH I, BOHR VA AND KOHN KW. (1991). Relationship between DNA cross-links, cell cycle, and apoptosis in Burkitt's lymphoma cell lines differing in sensitivity to nitrogen mustard. *Cancer Res.*, **51**, 6550-6557.

O'CONNOR PM, FERRIS DK, WHITE GA, PINES J, HUNTER T, LONGO DL AND KOHN KW. (1992). Relationship between cdc2 kinase, DNA cross-linking, and cell cycle perturbations induced by nitrogen mustard. *Cell Growth Different.*, **3**, 43-52.

O'CONNOR PM, FERRIS DK, PAGANO M, DRAETTA G, PINES J, HUNTER T, LONGO DL AND KOHN KW. (1993*a*). G2 delay induced by nitrogen mustard in human cells affects cyclin A/cdk2 and cyclin B1/cdc2-kinase complexes differently. *J. Biol. Chem.*, **268**, 8298-9303.

O'CONNOR PM, JACKMAN J, JONDLE D, BHATIA K, MAGRATH I AND KOHN KW. (1993*b*). Role of the p53 tumor suppressor in cell cycle arrest and radiosensitivity of Burkitt's lymphoma cell lines. *Cancer Res.* **53**, 4776-4780.

ORMEROD MG, ORR RM AND PEACOCK JH. (1994). The role of apoptosis in cell killing by cisplatin: a flow cytometric study. *Br. J. Cancer*, **69**, 93-100.

OSHIMA J, STEINMANN KE, CAMPISI J AND SCHLEGEL R. (1993). Modulation of cell growth, $p34^{cdc2}$ and cyclin A levels by SV-40 large T antigen. *Oncogene*, **8**, 2987-2993.

PINES J AND HUNTER T. (1989). Isolation of a human cyclin cDNA: evidence for cyclin mRNA and protein regulation in the cell cycle and for interaction with $p34^{cdc2}$. *Cell*, **58**, 833-846.

POWELL SN, DEFRANK JS, CONNELL P, EOGAN M, PREFFER F, DOMBKOWSKI D, TANG W AND FRIEND S. (1995). Differential sensitivity of $p53^{(-)}$ and $p53^{(+)}$ cells to caffeine-induced radiosensitization and override of G2 delay. *Cancer Res.*, **55**, 1643-1648.

RADFORD IR, MURPHY TK, RADLEY JM AND ELLIS SL. (1994). Radiation response of mouse lymphoid and myeloid cell lines. Part II. Apoptotic death is shown by all lines examined. *Int. J. Radiat. Biol.*, **65**, 217-227.

ROSENBLATT J, GU Y AND MORGAN DO. (1992). Human cyclin-dependent kinase 2 is activated during the S and G2 phases on the cell cycle and associates with cyclin A. *Proc. Natl Acad. Sci. USA*, **89**, 2824-2828.

RUBIN LL, PHILPOTT KL AND BROOKS SF. (1993). The cell cycle and cell death. *Curr. Biol.*, **3**, 391-394.

RUSSELL KJ, WIENS LW, DEMERS GW, GALLOWAY DA, PLON SE AND GROUDINE M. (1995). Abrogation of the G2 checkpoint results in differential radiosensitization of G1 checkpoint-deficient and G1 checkpoint-competent cells. *Cancer Res.*, **55**, 1639-1642.

SHI L, NISHIOKA WK, TH NG J, BRADBURY M, LITCHFIELD DW AND GREENBERG AH. (1994). Premature $p34^{cdc2}$ activation required for apoptosis. *Science*, **263**, 1143-1145.

SKLADANOWSKI A AND KONOPA J. (1993). Adriamycin and daunomycin induce programmed cell death (apoptosis) in tumour cells. *Biochem. Pharmacol.*, **46**, 375-382.

SKLADANOWSKI A, PLISOV SY, KONOPA J AND LARSEN AK. (1996). Inhibition of DNA topoisomerase II by imidazoacridinones, new antineoplastic agents with strong activity against solid tumours. *Mol. Pharmacol.*, **49**, (in press).

SLICHENMYER WJ, NELSON WG, SLEBOS RJ AND KASTAN MB. (1993). Loss of p53-associated G1 checkpoint does not decrease cell survival following DNA damage. *Cancer Res.*, **53**, 4164-4168.

SORENSON CM AND EASTMAN A. (1988*a*). Influence of *cis*-diamminedichloroplatinum(II) on DNA synthesis and cell cycle progression in excision repair proficient and deficient Chinese hamster ovary cells. *Cancer Res.*, **48**, 6703-6707.

SORENSON CM AND EASTMAN A. (1988*b*). Mechanism of *cis*-diamminedichloroplatinum(II)-induced cytotoxicity: role of G2 arrest and DNA double-strand breaks. *Cancer Res.*, **48**, 4484-4488.

SORENSON CM, BARRY MA AND EASTMAN A. (1990). Analysis of events associated with cell cycle arrest in G2 phase and cell death induced by cisplatin. *J. Natl Cancer Inst.*, **82**, 749-755.

STEINMANN KE, BELINSKY GS, LEE D AND SCHLEGEL R. (1991). Chemically induced premature mitosis: differential response in rodent and human cells and the relationship to cyclin B synthesis and p34$^{cdc2}$/cyclin B complex formation. *Proc. Natl Acad. Sci. USA*, **88**, 6843–6847.

STEINMANN KE, PEI XF, STÖPPLER H, SCHLEGEL R AND SCHLEGEL R. (1994). Elevated expression and activity of mitotic regulatory proteins in human papillomavirus-immortalized keratinocytes. *Oncogene*, **9**, 287–294.

TAM SW AND SCHLEGEL R. (1992). Staurosporine overrides checkpoints for mitotic onset in BHK cells. *Cell Growth Diff.*, **3**, 811–817.

TOUNEKTI O, PRON G, BELEHRADEK JJ AND MIR LM. (1993). Bleomycin, an apoptosis-mimetic drug that induces two types of cell death depending on the number of molecules internalized. *Cancer Res.*, **53**, 5462–5469.

TSAO Y-P, D'ARPA P AND LIU LF. (1992). The involvement of active DNA synthesis in camptothecin-induced G2 arrest: altered regulation of p34$^{cdc2}$/cyclin B. *Cancer Res.*, **52**, 1823–1829.

VINDELØV LL, CHRISTENSEN IJ AND NISSEN NI.(1992). A detergent-trypsin method for the preparation of nuclei for flow cytometric DNA analysis. *Cytometry*, **3**, 323–327.

WARTERS RL. (1992). Radiation-induced apoptosis in a murine T-cell hybridoma. *Cancer Res.*, **52**, 883–890.

WEINERT TA AND LYDALL D. (1993). Cell cycle checkpoints, genetic instability and cancer. *Semin. Cancer Biol.*, **4**, 129–140.

YAMAGISHI T, NAKAIKE S, NANAUMI K, OTOMO S AND TSUKAGOSHI S. (1993). The effect of NC-190, a novel antitumor compound, on the cell-cycle progression of HeLa S3 cells. *Cancer Chemother. Pharmacol.*, **32**, 249–254.